# A Preliminary Investigation of the Views of People With Parkinson's (With and Without Psychosis) and Caregivers on Participating in Clinical Trials During the Covid-19 Pandemic: An Online Survey

**DOI:** 10.3389/fpsyt.2020.602480

**Published:** 2020-12-23

**Authors:** Katie McGoohan, Anneesa Amjad, Natasha Ratcliffe, Sagnik Bhattacharyya, Gillian Granville, Matthew Sullivan, Lesley Gosden, Dag Aarsland, K. Ray Chaudhuri, Dominic ffytche, Clive Ballard, Latha Velayudhan

**Affiliations:** ^1^Department of Old Age Psychiatry, Institute of Psychiatry, Psychology and Neuroscience, King's College London, London, United Kingdom; ^2^Parkinson's UK, London, United Kingdom; ^3^Department of Psychosis Studies, Institute of Psychiatry, Psychology and Neuroscience, King's College London, London, United Kingdom; ^4^Centre for Age-Related Medicine, Stavanger University Hospital, Stavanger, Norway; ^5^Parkinson Foundation International Centre of Excellence, King's College Hospital and Kings College London, London, United Kingdom; ^6^University of Exeter Medical School, St Luke's Campus, Exeter, United Kingdom; ^7^South London and Maudsley National Health Service Foundation Trust, London, United Kingdom

**Keywords:** hallucinations and delusions, patient and public involvement (PPI), COVID-19, clinical trial, psychosis, survey, Parkinson's disease

## Abstract

**Background:** The coronavirus pandemic is having a profound impact on non-COVID-19 related research, including the delivery of clinical trials for patients with Parkinson's disease.

**Objectives:** A preliminary investigation to explore the views of Parkinson's disease (PD) patients, with and without experience of psychosis symptoms, and carers on the resumption of clinical research and adaptations to trials in light of COVID-19.

**Methods:** An anonymous self-administered online survey was completed by 30 PD patients and six family members/carers via the Parkinson's UK Research Support Network to explore current perceptions on taking part in PD research and how a planned clinical trial for psychosis in PD may be adapted so participants feel safe.

**Results:** Ninety-one percent of respondents were enthusiastic about the continuation of non-COVID-19 related research as long as certain safety measures were in place. Ninety-four percent stated that they would be happy to complete assessments virtually. However, they noted that care should be taken to ensure that this does not exclude participants, particularly those with more advanced PD who may require assistance using portable electronic devices. Regular and supportive communication from the research team was also seen as important for maintaining the psychological well-being of participants while taking part in the trial.

**Conclusions:** In the era of COVID-19 pandemic, standard approaches will have to be modified and rapid adoption of virtual assessments will be critical for the continuation of clinical research. It is important that alongside the traditional methods, new tools are developed, and older ones validated for virtual assessments, to allow safe and comprehensive assessments vital for ongoing research in people with Parkinson's.

## Introduction

The current coronavirus disease 2019 (COVID-19) pandemic is having a huge impact on healthcare systems and broader society across the world. Patients with chronic conditions are being significantly affected by loss of social contact, constraints on movement and disruption to access to both urgent and routine healthcare, with many outpatient appointments being canceled or postponed (1, 2). While implementation of telemedicine has increased dramatically (3), allowing for the continuation of ongoing care and remote monitoring (2, 4, 5) all of these factors raise some serious concerns for the health and well-being of patients with Parkinson's disease (PD) (6).

Although research has played an important part in our response to COVID-19, the outbreak has also had an impact on the conduct and delivery of non-COVID-19 related research. Most other research has been paused or significantly reduced, including many clinical trials for PD (7, 8). While this is understandable due to concern for the safety of trial participants and research staff, it is vital that trials resume in order to meet the unmet clinical needs for people with PD (9).

Parkinson's disease psychosis (PDP) refers to the range of hallucinations and delusions that occur in PD (10). Their prevalence increases with illness duration, with most patients eventually developing such symptoms (11). Although there are a number of clinical options available for treating PDP, they are either not very effective or require specialized monitoring of side effects (12). It is vitally important that promising interventions continue to be tested in the form of a clinical trial. In light of the current pandemic, changes to the traditional methods used for delivering research are therefore required so that research can resume as soon as it is safe to do so (13).

In considering these changes, it is vital that researchers work with patients and the public to understand their views on research participation during pandemic times and to ensure that trial adaptations are practical for future study participants. Patient and public involvement (PPI) is defined as research being carried out “with” or “by” members of the public rather than “to,” “about,” or “for” them (14). It is an essential activity in all stages of the research process and ensures the acceptability and relevance of research (15). This study reports the results of an online survey that was developed together with a group of PPI advisors to explore PD patients' views on taking part in research during the current pandemic and how a planned clinical trial for people with PDP may be best adapted so that participants feel safe. To ensure that the results of the survey were reflective of the participants who would be taking part in the planned clinical trial, we were specifically interested in recruiting people with experience of psychosis symptoms. However, as the results of this survey would be of interest to a much wider audience (e.g., those conducting research with patients with other neuropsychiatric disorders or older adults more generally), the survey was open to anyone with PD.

## Methods

Views were gathered using an online survey, which included a mixture of closed- and open-ended questions, the full details of which are provided in the Supplementary Material. The survey was co-created with three patient advisors (whose involvement was facilitated by Parkinson's UK) to ensure that the questions being asked in the survey were relevant to and informed by the perspective of people with PD. The patient advisors provided input on the questions, response options and format of the initial draft of the survey. The survey consisted of 19 questions: six questions gathering details about survey respondents, seven questions related to general perceptions about taking part in research at the current time, followed by five more specific questions about adaptations to the planned clinical trial investigating psychosis in PD. It should be noted that for some of the questions, multiple responses could be selected (see questions with “Tick all that apply” statement in Supplementary Material). The final question was a free text option asking what physical or psychological support would help people with PD take part in research at this time.

An invitation to participate was distributed by Parkinson's UK to their Research Support Network—an online network that brings together people driven to help find a cure and better treatments for PD (16). The survey was open to (inclusion criteria) anyone affected by Parkinson's- including partners, carers and family members of those with the condition, and people who had experience of hallucinations and/or delusions were especially encouraged to participate to ensure that any changes being made to the planned clinical trial were inclusive and accessible to future participants. The questionnaire was in English and sent out via email, so excluded those who were not-fluent in English or who did not have access to computers or have an email address. Caregivers or family members were able to complete the survey on behalf of a person affected by PD (e.g., the person with PD was unable to complete the survey themselves). People expressed their interest to Parkinson's UK and were then sent a link to the survey, along with a plain English summary of the trial. The survey was administered using SmartSurvey, an online survey software and questionnaire tool. As any adaptations to the planned clinical trial were required to be processed within a timely manner, the survey was open to responses from 26 June to 6 July 2020.

Survey responses were fully anonymized and no identifiable information was collected. The survey was conducted as a PPI activity therefore no ethical approval was required. Informed consent was obtained from all participants.

Simple statistical summaries were generated for the closed form responses to each survey item. Since responses to the open-ended questions were fairly succinct, no formal qualitative analysis or prespecified framework were imposed on the open response data. To aid interpretation of the quantitative results, free-text responses were grouped into categories and classified as being positive, negative or neutral in tone (for example, whether respondents were generally positive, had reservations or were reluctant about the continuation of research in their comments).

## Results

Thirty people with PD (83%) and six carers, partners or family members (17%) completed the survey. Fourteen (47%) of the respondents with PD had experience of psychosis symptoms: five had experience of hallucinations, two of delusions, and seven of both symptoms of psychosis. All six carers lived with someone who had experience of psychosis symptoms: three each with experience of hallucinations alone and both hallucinations and delusions. Table 1 details characteristics of the survey respondents and Table 2 the main results of the closed form responses.

**Table 1:** Details of survey respondents: PD participants, and carers/partners/family members on behalf of PD participants.

		**PD participants, *n* = 30 (%)**	**Carers, *n* = 6 (%)**
Time since PD diagnosis	Within the last year	1 (3)	0 (0)
	1–5 years	12 (40)	1 (17)
	5–10 years	8 (27)	2 (33)
	10–15 years	3 (10)	2 (33)
	More than 15 years	6 (20)	1 (17)
Experience of psychotic symptoms^*^	Experience of hallucinations and delusions	7 (24)	3 (50)
	Experience of hallucinations	5 (17)	3 (50)
	Experience of delusions	2 (7)	0 (0)
	No experience of hallucinations or delusions	15 (52)	0 (0)
Living arrangements	Living with partner	25 (83)	5 (83)
	Living with family/friends	1 (3)	0 (0)
	Living on their own	4 (13)	0 (0)
	Other (live in carer)	0 (0)	1 (17)
Previous participation in research	No previous participation	12 (40)	3 (50)
	Participation in online research	17 (57)	2 (33)
	Participation in a clinical trial	3 (10)	1 (17)
	Other	1 (3)	0 (0)

* *Missing data from one participant with PD*.

*PD, Parkinson's Disease*.

**Table 2:** Survey responses to closed form questions from PD participants with and without experience of psychosis symptoms and from carers, family members or partners.

**Survey questions**	**Response options**	PD participant with psychosis symptoms, *n* = 14^*^ (%)	PD participant without psychosis symptoms, *N* = 15^*^ (%)	**Carers, family members or partners, *n* = 6 (%)**
Preference for study visit location	More comfortable taking part in research from home	4 (29)	4 (27)	5 (83)
	More comfortable taking part in research that involved a visit to a clinical setting	2 (14)	1 (7)	0 (0)
	Comfortable either way	8 (57)	10 (67)	1 (17)
	Not comfortable either way	0 (0)	0 (0)	0 (0)
What might help a home visit from a researcher feel safe	PPE for the researcher	9 (64)	11 (73)	4 (67)
	PPE for participant	8 (57)	8 (53)	1 (17)
	The researcher traveling by car (not using public transport)	8 (57)	9 (60)	4 (67)
	The researcher having regular tests for COVID-19	8 (57)	9 (60)	5 (83)
Maximum length of time for home visit	1 h	1 (7)	7 (47)	3 (50)
	2 h	11 (79)	5 (33)	3 (50)
	3 h	1 (7)	1 (7)	0 (0)
	4 h	0 (0)	0 (0)	0 (0)
	5 h	1 (7)	2 (13)	0 (0)
What might help a study visit in a clinical setting feel safe	PPE for the researcher	10 (71)	10 (67)	4 (67)
	Participants being required to wear a mask	9 (64)	10 (67)	3 (50)
	Participants being required to use their own personal transport or being offered a taxi	12 (86)	9 (60)	4 (67)
	Thorough cleaning of assessment rooms in between participants	9 (64)	8 (53)	3 (50)
Maximum length of time for visit to clinical setting	1 h	0 (0)	5 (33)	3 (50)
	2 h	12 (86)	6 (40)	3 (50)
	3 h	0 (0)	1 (7)	0 (0)
	4 h	1 (7)	0 (0)	0 (0)
	5 h	1 (7)	3 (20)	0 (0)
Willingness to complete study assessments virtually	Yes	13 (93)	15 (100)	5 (83)
	No	1 (7)	0 (0)	1 (17)
Willingness to take a finger-prick blood test at home	Yes	14 (100)	14 (93)	6 (100)
	Not sure	0 (0)	1 (7)	0 (0)
Willingness to take a pregnancy test at home	Yes	2 (14)	2 (13)	1 (17)
	No	2 (6)	0 (0)	0 (0)
	Not applicable	10 (71)	13 (87)	5 (83)
Willingness to track of study drug compliance at home	Yes	14 (100)	14 (93)	5 (83)
	Not sure	0 (0)	1 (7)	1 (17)

* *Missing data from one participant with PD who did not respond to the question asking whether they had experience of hallucinations and/or delusions*.

*PPE, Personal protective equipment*.

### General Feelings on Taking Part in Research

When asked what their feelings were about taking part in research at present, or in the near future, given the current COVID-19 pandemic, the majority of respondents (69%) were positive about the continuation of non-COVID-19 related research as long as it was safe to do so. This was supported by numerous comments about the importance of research:

“*It is important to carry on with research as life goes on research is still necessary to help with finding a cure. Without this there will not be any answers. Whatever we come up against we all have to deal with.”*
“*I am keen to take part in any trials regarding PD. There is no cure, that doesn't mean we shouldn't be looking for one*.”
“*I think research is very important and if it can be carried out in a safe way I'm happy to participate.”*
“*I am quite happy to take part in research if it may help my wife and the many others suffering from Parkinson's.”*

Some respondents (22%) were positive but had reservations or requirements for participation to feel safe:

“*I have no objection to taking part in clinical research, providing my participation takes place within the safe rules governing COVID-19.”*
“*I have to take sensible precautions.”*
“*I would like to be tested to see if I have had COVID-19 before taking part.”*

It was also clear from some of the comments that participants were particularly reluctant to attend hospital visits, preferring research activities to be conducted virtually:

“*I feel comfortable with taking part in PD related research activities but would prefer not to have to visit hospitals and clinics*.”
“*I am happy to do on-line research but will not attend either a hospital or a face to face meeting of any type.”*

Only a couple of respondents (6%), both of whom had experience of psychosis symptoms, expressed reluctance to participate in research at the present time.

### Preferred Location and Length of Time for Visits

When asked whether respondents would feel more comfortable taking part in research if they did not have to visit a clinical setting, over 50% said they would feel comfortable either way, 39% said they would feel more comfortable taking part in research from home, 8% said they would feel more comfortable taking part in research that involved a clinical setting and no respondents selected “I would not feel comfortable either way.” Carers were more inclined (83%) toward study visits taking place at their home compared to PD participants (27%). These respondents were often caring for people with complex needs and did not think it appropriate to visit other settings.

In response to the question asking how they would feel about a researcher visiting their home to conduct study visits, 69% of respondents expressed that they would be agreeable to a researcher conducting a home visit, and a further 22% stated that they would be willing as long as appropriate safety measures were taken. This was backed up by open-text comments throughout the survey:

“*I am happy to do that. As long as all precautionary measures are put in place on both sides.”*
“*As long as exemplary COVID-19 precautions are taken—not a problem for me.”*

To help make a home visit from a researcher feel safe, many respondents selected that they would like to see all the multiple-choice options that we provided in the survey being applied: (1) personal protective equipment (PPE) for the researcher (67%), (2) the researcher having regular tests for COVID-19 (64%), (3) the researcher traveling by car (not using public transport) (61%), and (4) PPE for the participant (47%). Further suggestions included meetings to be held outdoors (for example, in participant's gardens) and maintaining a safe distance between the participant and researcher.

Similarly, when asked what would help to make a study visit at a local hospital feel safe, most respondents selected all of the multiple-choice options that we provided in the survey: (1) participants being required to use their own personal transport or being offered a taxi (72%), (2) researchers wearing personal protective equipment (67%), (3) participants being required to wear a mask (61%), and (4) thorough cleaning of assessment rooms in between participants (58%). In addition, most respondents were willing for both home (67%) and hospital (75%) visits to last 2 hours or more.

Only 17% of carers and 44% of PD participants with experience of delusions felt that participants should be required to wear PPE in their own homes, compared to 60% of PD participants without symptoms of delusions (no experience of psychosis or symptoms of hallucinations only). PD participants with experience of delusions were also more likely to require researchers (89%) and participants (100%) to use personal transport when traveling to study visits compared to participants without these symptoms (45 and 60% respectively).

### Virtual Assessments

A breakdown of the types of assessment that respondents would be amenable to completing virtually as part of the planned clinical trial is provided in Table 3. Only two respondents, both of whom had experience of psychosis, said they would not feel comfortable carrying out study assessments virtually.

**Table 3:** Study assessments that respondents would be willing to complete virtually or whether they would prefer face to face.

	**Telephone, *N* = 36 (%)**	**Video call, *n* = 36 (%)**	**Online survey, *n* = 36 (%)**	**Prefer face to face, *n* = 36 (%)**
Consent	32 (89)	24 (67)	24 (67)	3 (8)
Medical history and current medications	30 (83)	23 (64)	24 (67)	5 (14)
Sociodemographic information	31 (86)	22 (61)	25 (69)	3 (8)
Adverse events	27 (75)	22 (61)	21 (58)	7 (19)
Motor symptoms of PD	28 (78)	24 (67)	24 (67)	4 (11)
Non-motor symptoms of PD	27 (75)	23 (64)	26 (72)	5 (14)
Quality of life	29 (81)	21 (58)	24 (67)	6 (17)

*PD, Parkinson's Disease*.

Telephone calls was the preferred method for remote follow-up compared to video call or online surveys. Reasons for this varied in the open text comments but were mainly driven by issues with connectivity and feelings of discomfort toward the use of internet-based technology:

“*On the whole I don't like video calls as I feel self-conscious, but I would be prepared to overcome this.”*
“*I am not comfortable using my computer, so I prefer to fill in a real form than a virtual one.”*

When asked what would make virtual assessments easier to complete, respondents noted that questionnaires should be easy to understand and come with clear guidance and instructions. Support from a family member or carer and flexibility in the way that assessments could be completed (i.e., ability to complete in several sittings), were also seen as important in the comments:

“*My mother would need assistance of a carer to complete any assessment and responses would be by 3rd party from the carers.”*

Almost all respondents were willing to take a finger-prick home blood test (as a remote alternative to venepuncture) (97%) as long as they had clear instructions and, if required, the support of a carer. Respondents were also willing to monitor compliance with study drugs (i.e., pill counts (94%)) at home. Suggestions for what would make this easier to complete included the use of an online diary and pill dispensers.

### Additional Support

The final question in the survey asked what other physical or psychological support would help people with PD take part in a research study at this time. Supportive and regular contact from research teams, preferably with a designated contact for the length of the study, was frequently suggested by respondents. For those with psychosis symptoms, importance was also given in the comments to the emotional support required when answering difficult questions relating to their symptoms:

“*Dealing with distress caused by recalling upsetting episodes of hallucinations.”*

Other recommendations included maintained engagement with the progress of the trial and the requirement for peer or carer support to assist with participation in the trial, particularly when considering remote assessments that require use of technology.

## Discussion

Following the Guidance for Reporting Involvement of Patients and Public, 2 (GRIPP2; see Supplementary Material), this paper highlights the importance of PPI in ensuring that any changes that are made to the way that we deliver research for people with PD in a world with COVID-19 are acceptable to those who will be participating (17).

Our survey findings suggest that despite the current pandemic, PD patients and their carers see the importance of research and remain enthusiastic about participation. Although a small proportion of respondents in this survey was anxious about taking part in research in the short term, most respondents were comfortable for researchers to conduct face to face study assessments, as long as adequate safety precautions were in place.

Much of the open-ended data provided by the respondents emphasized the importance of taking a flexible approach to research. Following the COVID-19 pandemic, there has been rapid growth and development in the area of telemedicine and digital healthcare, which can be easily adapted for use in PD research for the evaluation of both motor and non-motor symptoms (2). While virtual follow-up may not be possible for all types of assessment [for example, physical examinations (18)], it may help reduce the length of time required for in-person visits, thereby potentially reducing the likelihood of infection acquired during the study visit (3, 19). Whilst almost all respondents reported that they would feel comfortable carrying out some of the study assessments and questionnaires virtually, care would be needed to ensure that we do not exclude participants, particularly those without the skills or support to use portable electronic devices (20). Researchers should therefore be prepared to provide additional information and one-to-one support to ensure that participants feel comfortable. Providing options in the way that participants can interact with researchers during visits and throughout the study was identified as being of the utmost importance so that participants felt supported. Virtual assessments in the clinical trial may therefore be used as part of a flexible package of follow-up methods, alongside traditional telephone and face to face approaches (21).

Although the sample size of our survey was small, this is likely attributable to our short period of data collection and request for people who had experience of psychosis. Nevertheless, this was important to ensure that some of the views were reflective of the sample who will be taking part in the planned clinical trial for psychosis in PD. Psychosis typically occurs in the later stages of PD, with risk factors including older age, increased duration and severity of PD, and significant psychiatric or medical comorbidity (11, 22). While there is currently no evidence to suggest that those with PD are at increased risk of contracting coronavirus (9, 23), advanced PD patients [for whom the prevalence of PDP ranges from 20 to 70% (11)] may be more susceptible and at greater risk for respiratory complications after a COVID-19 infection (24). Despite this, attitudes toward the continuation of research in the current climate were equally positive among participants both with, and without, experience of psychosis. Analysis between the two groups showed that those with experience of PDP (particularly carers and family members) were more inclined toward face to face visits taking place in a home setting, and open responses emphasized the requirement for ongoing physical and psychological support from carers and the research team, however, the sample size is too small to draw conclusions about such patterns.

All carers, partners and family members who participated in the survey lived with someone who had experience of psychosis symptoms. It is therefore not surprising that carers preferred study visits to take place in an environment in which the participant is familiar. Although our numbers were too small to demonstrate differences between the groups with any certainty, it does seem that carers and participants with experience of delusions were also less likely to suggest that participants wear PPE during a home visit. While the benefits of PPE are clear, we must be aware of the potentially disorientating impact that wearing PPE may have on patients, particularly those suffering from symptoms of psychosis. It is therefore important that researchers provide participants with additional information on what to expect during study participation, including clear information about any COVID safety measures that are in place, prior to the study visit. Not only will this provide reassurance, but it may also help reduce any anxiety and enable a more informed decision about research participation.

In the early stages of PD, hallucinations typically occur with insight initially preserved, whereas in the later disease stages, patients might not recognize the hallucinations as unreal anymore due to the onset of false beliefs (delusions) (10, 12). Given this progression, one might anticipate respondents with experience of delusions to have impaired insight compared to those with no experience of psychosis symptoms or experience of hallucinations only. Interestingly however, this did not appear to be the case among respondents of this survey. The only difference in responses between PD participants with and without symptoms of delusions was the requirement for researchers and participants to use their own personal transport or taxis when traveling to study visits, perhaps showing an increased awareness of the risks associated with COVID-19 among this more advanced PD population group. However, such an interpretation is tentative because of the small size of the sub-groups.

### Limitations

Only 11% of participants had previous experience of a clinical trial, so may not be familiar with the types of procedures and assessments that this would typically involve. However, the fact that these participants still expressed their interest in taking part in future trials and research is noteworthy. It is also worth mentioning that self-report of psychotic symptoms would depend on respondent's insight and willingness to share such information, hence could have been under-reported. The results of this survey should also be considered in the context of a group with a specific interest in PD research. Despite this, it was clear from the responses that members of the Research Support Network used their knowledge of other people with PD as well as their own experiences, and their views were invaluable in informing how best to adapt the clinical trial. PPI members will continue to inform and improve participation in this research through their involvement with the trial's Patient Advisory Group and Trial Steering Committee.

Although our sample size was small, this survey was conducted with a fairly unique population, and highlights for the first time the views of PD patients and caregivers on taking part in research during the COVID-19 pandemic. The sample size was also deemed appropriate because of the exploratory nature of this research and the focus on identifying initial attitudes about the topic. While this study focused on patients with PD, many of the broader insights and recommendations will also be applicable to those involved in the design and delivery of clinical research with patients with other neuropsychiatric disorders as well an older adult population more generally.

### Future Research

Although the sample size was deemed appropriate due to the exploratory nature of this research, a larger sample may have identified additional viewpoints or provided more nuanced explanations for PD patients and caregivers attitudes toward the continuation of research during pandemic times. It would therefore be useful to conduct a broader survey on the topic of adapting research in light of COVID-19, helping ensure that adaptations made to clinical studies are informed by the needs of people affected by Parkinson's. Future research would benefit from collecting data on current neurological, psychiatric and cognitive status, treatment and caregiver burden to determine whether these symptoms would influence responses. Researchers might also consider asking questions about all the types of devices participants have access to in their home and/or their technical literacy. It would be useful for upcoming clinical trials in PD to add a qualitative component to their study to capture the views and opinions of participants who are taking part in research on any ethical challenges or other barriers encountered, especially during the period of the pandemic.

## Conclusion

Non-COVID-19 related research remains of critical importance and must not be neglected. Although patients with PD remain enthusiastic about participation in research, a flexible approach to the way that we redesign and deliver our studies in a world where travel and face to face contact are restricted is required. New tools should be developed and existing tools should be validated for virtual use as a matter of urgency to ensure research can continue to be delivered during the COVID-19 pandemic.

## Data Availability Statement

The raw data supporting the conclusions of this article will be made available by the authors, without undue reservation.

## Ethics Statement

Ethical review and approval was not required for the study on human participants in accordance with the local legislation and institutional requirements. The patients/participants provided their written informed consent to participate in this study.

## Author Contributions

LV, SB, KM, NR, and AA: conceptualization. KM, AA, NR, LG, MS, GG, LV, and SB: survey design. AA and KM: analysis and interpretation of data. KM, LV, AA, NR, GG, and MS: writing—original draft. KM, AA, NR, MS, GG, LV, DA, KC, SB, DF, and CB: writing—revising and providing the final approval of work. All authors contributed to the article and approved the submitted version.

## Conflict of Interest

The authors declare that the research was conducted in the absence of any commercial or financial relationships that could be construed as a potential conflict of interest.
