# Supplementary material for: A Preliminary Investigation of the Views of People With Parkinson's (With and Without Psychosis) and Caregivers on Participating in Clinical Trials During the Covid-19 Pandemic: An Online Survey
Source: Front Psychiatry. 2020 Dec 23;11:602480. doi: 10.3389/fpsyt.2020.602480 (PMC7785792; doi:10.3389/fpsyt.2020.602480)
Supplement: Supplementary file 1 [file Data_Sheet_1.docx]

**Supplementary Material**

**S1: Survey questions**

**Details of survey respondents: Parkinson’s disease patients**

1. Consent statement ‘By proceeding with the survey and clicking 'I agree' below, you consent to your data being used as described above.’ I agree

2. What is your association with Parkinson's? I have Parkinson’s/ I am a carer, partner or family member of someone with Parkinson's

3. When were you diagnosed with Parkinson's? Within the last year/ 1 - 5 years ago/

5 - 10 years ago/ 10 - 15 years ago/ More than 15 years ago

4. What are your living arrangements? I live with my partner/ I live with family/friends/ I live on my own/ I live in a care home/ Other (free text)

5. Do you experience hallucinations (seeing things or hearing voices that are not there) or delusions (false beliefs) as part of your Parkinson's? Yes, I experience hallucinations and delusions/ Yes, I experience hallucinations/ Yes, I experience delusions/ No, I don't experience hallucination or delusions

 6. Have you participated in research before? No/ Yes, I've participated in online research (such as questionnaires)/ Yes, I've participated in a clinical trial/ Other (free text)

**Details of Survey Respondents: Caregivers**

1. Consent statement ‘By proceeding with the survey and clicking 'I agree' below, you consent to your data being used as described above.’ I agree

2. What is your association with Parkinson's? I have Parkinson’s/ I am a carer, partner or family member of someone with Parkinson's

3. When was the person with Parkinson's diagnosed? Within the last year/ 1 - 5 years ago/

5 - 10 years ago/ 10 - 15 years ago/ More than 15 years ago

4. What are the person with Parkinson's living arrangements? They live with their partner/ They live with their family/friends/ They live on their own/ They live in a care home/

Other (free text)

5. Do they experience hallucinations (seeing things or hearing voices that are not there) or delusions (false beliefs) as part of their Parkinson's? Yes, they experience hallucinations and delusions/ Yes, they experience hallucinations/ Yes, they experience delusions/ No, they don't experience hallucination or delusions

6. Have they participated in research before? No/ Yes, they've participated in online research (such as questionnaires)/ Yes, they've participated in a clinical trial/ Other (free text)

**General perceptions about taking part in research at the current time (all respondents)**

7. What are your feelings about taking part in research at present, or in the near future, given the current Covid-19 pandemic? (free text)

 8. Would you feel more comfortable taking part in research if you did not have to visit a clinical setting (such as a hospital or university)? I would feel more comfortable taking part in research from home/ I would feel more comfortable taking part in research that involved a visit to a clinical setting/ I would feel comfortable either way/ I would not feel comfortable either way

If you were to take part in a research study at this time, how would you feel about a researcher visiting your home to conduct the study visits? (free text)

9. What would help to make a home visit from a researcher feel safe? Tick all that apply. Personal protective equipment for the researcher/ Personal protective equipment for you/ The researcher travelling by car (not using public transport)/ The researcher having regular tests for Covid-19/ Other (free text)

10. What is the maximum length of time that a home visit should last? 1 hour/ 2 hours/ 3 hours/ 4 hours/ 5 hours/ Comments (free text)

11. If you were to take part in a research study at this time that required you to visit a local hospital for the study assessments, what would help this feel safe? Tick all that apply. Researchers wearing personal protective equipment/ Participants being required to wear a mask/ Participants being required to use their own personal transport or being offered a taxi/ Thorough cleaning of assessment rooms in between participants/ Other (free text)

12. What is the maximum length of time that a visit to a hospital for assessments should last? 1 hour/ 2 hours/ 3 hours/ 4 hours/ 5 hours/ Comments (free text)

13. Would you feel comfortable carrying out some of the study assessments using a computer, smartphone or tablet device? Yes/ No/ Not sure

**Specific questions about adaptions to the planned clinical trial (all respondents)**

14. We want to minimise the length of time taken for face-to-face visits (at home or in the hospital). Which of the following assessments would you be happy to complete virtually (i.e. over the phone, video-call or online survey)? Please tick all that apply.

Providing consent to take part in the study. I'd be happy to do this over the phone/ I'd be happy to do this by video call/ I'd be happy to do this via an online survey/ I'd prefer to do this face-to-face

Providing information about your medical history and current medications. I'd be happy to do this over the phone/ I'd be happy to do this by video call/ I'd be happy to do this via an online survey/ I'd prefer to do this face-to-face

Providing general information such as your age, gender, education. I'd be happy to do this over the phone/ I'd be happy to do this by video call/ I'd be happy to do this via an online survey/ I'd prefer to do this face-to-face

Reporting any side effects that you may be experiencing from the study medication. I'd be happy to do this over the phone/ I'd be happy to do this by video call/ I'd be happy to do this via an online survey/ I'd prefer to do this face-to-face

Completing questionnaires on movement symptoms of Parkinson's. I'd be happy to do this over the phone/ I'd be happy to do this by video call/ I'd be happy to do this via an online survey/ I'd prefer to do this face-to-face

Completing questionnaires on non-movement symptoms of Parkinson's. I'd be happy to do this over the phone/ I'd be happy to do this by video call/ I'd be happy to do this via an online survey/ I'd prefer to do this face-to-face

Completing questionnaires on quality of life. I'd be happy to do this over the phone/ I'd be happy to do this by video call/ I'd be happy to do this via an online survey/ I'd prefer to do this face-to-face

15. What would make these assessments easier to complete virtually? (free text)

16. If you were taking part in the study, would you be happy to take a finger-prick blood test (similar to how people with diabetes check their blood sugar levels) at home? Yes/ No/ Not sure/ What would make it easier to do this? (free text)

17. If you were taking part in the study, would you be happy to take a pregnancy test (which involves taking a sample of urine) at home? Yes/ No/ Not sure/ Not applicable/ What would make it easier to do this? (free text)

18. If you were taking part in the study, would you be happy to keep track of how many of the study medication capsules you have taken and if any were missed at home? Yes/ No/ Not sure/ What would make it easier to do this? (free text)

**Other psychical or psychological support (all respondents)**

19. What other physical or psychological support do you think would help people with Parkinson's take part in a research study at this time? (free text)

**S2:** **GRIPP2 Checklist (long form)**

| Section and topic | Item | Reported |
| --- | --- | --- |
| Section 1: Abstract of paper | |  |
| 1a: Aim | Report the aim of the study | Yes |
| 1b: Methods | Describe the methods used by which patients and the public were involved | Yes |
| 1c: Results | Report the impacts and outcomes of PPI in the study | Yes |
| 1d:Conclusions | Summarise the main conclusions of the study | Yes |
| 1e: Keywords | Include PPI, “patient and public involvement,” or alternative terms as keywords | Yes |
| Section 2: Background to paper | |  |
| 2a: Definition | Report the definition of PPI used in the study and how it links to comparable studies | Yes |
| 2b: Theoretical underpinnings | Report the theoretical rationale and any theoretical influences relating to PPI in the study | n/a |
| 2c: Concepts and theory development | Report any conceptual models or influences used in the study | n/a |
| Section 3: Aims of paper | |  |
| 3: Aim | Report the aim of the study | Yes |
| Section 4: Methods of paper | |  |
| 4a: Design | Provide a clear description of methods by which patients and the public were involved | Yes |
| 4b: People involved | Provide a description of patients, carers, and the public involved with the PPI activity in the study | Yes |
| 4c: Stages of involvement | Report on how PPI is used at different stages of the study | Yes |
| 4d: Level or nature of involvement | Report the level or nature of PPI used at various stages of the study | Yes |
| Section 5: Capture or measurement of PPI impact | |  |
| 5a: Qualitative evidence of impact | If applicable, report the methods used to qualitatively explore the impact of PPI in the study | n/a |
| 5b: Quantitative evidence of impact | If applicable, report the methods used to quantitatively measure or assess the impact of PPI | n/a |
| 5c: Robustness of measure | If applicable, report the rigour of the method used to capture or measure the impact of PPI | n/a |
| Section 6: Economic assessment | |  |
| 6: Economic assessment | If applicable, report the method used for an economic assessment of PPI | n/a |
| Section 7: Study results | |  |
| 7a: Outcomes of PPI | Report the results of PPI in the study, including both positive and negative outcomes | Yes |
| 7b: Impacts of PPI | Report the positive and negative impacts that PPI has had on the research, the individuals involved (including patients and researchers), and wider impacts | Yes |
| 7c: Context of PPI | Report the influence of any contextual factors that enabled or hindered the process or impact of PPI | Yes |
| 7d: Process of PPI | Report the influence of any process factors, that enabled or hindered the impact of PPI | n/a |
| 7ei: Theory development | Report any conceptual or theoretical development in PPI that have emerged | n/a |
| 7eii: Theory development | Report evaluation of theoretical models, if any | n/a |
| 7f: Measurement | If applicable, report all aspects of instrument development and testing (eg, validity, reliability, feasibility, acceptability, responsiveness, interpretability, appropriateness, precision) | n/a |
| 7g: Economic assessment | Report any information on the costs or benefit of PPI | Yes |
| Section 8: Discussion and conclusions | |  |
| 8a: Outcomes | Comment on how PPI influenced the study overall. Describe positive and negative effects | Yes |
| 8b: Impacts | Comment on the different impacts of PPI identified in this study and how they contribute to new knowledge | Yes |
| 8c: Definition | Comment on the definition of PPI used (reported in the Background section) and whether or not you would suggest any changes | Yes |
| 8d: Theoretical underpinnings | Comment on any way your study adds to the theoretical development of PPI | n/a |
| 8e: Context | Comment on how context factors influenced PPI in the study | Yes |
| 8f: Process | Comment on how process factors influenced PPI in the study | n/a |
| 8g: Measurement and capture of PPI impact | If applicable, comment on how well PPI impact was evaluated or measured in the study | n/a |
| 8h: Economic assessment | If applicable, discuss any aspects of the economic cost or benefit of PPI, particularly any suggestions for future economic modelling. | n/a |
| 8i: Reflections/critical perspective | Comment critically on the study, reflecting on the things that went well and those that did not, so that others can learn from this study | Yes |
